# Quarterly Report of Ophthalmological and Otological Literature*Authors are requested to send a re-print of their papers, or the number of the journal in which they appear, to Dr. E. J. Gardiner, 170 State st., Chicago, Ill.

**Published:** 1881-06

**Authors:** E. J. Gardiner

**Affiliations:** Chicago


### Summary.

*Collaborators:*

DR. L. W. CASE, DR. R. TILLEY,
DR. BYRON W. GRIFFIN, DR. W. L. DORLAND,
DR. H. D. VALIN.

QUARTERLY ABSTRACT OF OPHTHALMOLOGICAL AND OTOLOGICAL LITERATURE.* By Dr. E. J. GARDINER, Chicago.

THE APPLICATION OF ELECTROLYSIS IN OPHTHALMIC THERAPEUTICS. By A. Nieden, M.D., of Bochum.—(*Arch. of Opth., Vol.* X., *No.* 1.)

N. highly recommends the use of electrolysis in the treatment of angioma of the lids, and reports several cases cured by its application. The portable, constant apparatus made by Krüger, of Berlin, after the model Spamer, was used. The needle of the positive pole should be made of platinum, steel needles having the disagreeable feature of becoming rapidly oxidized, and discoloring the tissues by the intermingling of the products of oxidation with them. "The needles are inserted at the places appearing most desirable for the first attack; in small tumors, one for each pole; in larger ones, two or more for the negative pole." His results have been very satisfactory.

CONTRIBUTIONS TO THE KNOWLEDGE OF THE CONGENITAL DISPLACEMEMT OF THE LENS. By Dr. H. E. D'Oench, of St. Louis.—(*Arch. of Oph., Vol.* X., *No.* 1.)

After reporting the case of a girl, eight years old, in whose right eye there was found congenital dislocation of the lens, out-

* Authors are requested to send a re-print of their papers, or the number of the journal in which they appear, to Dr. E. J. Gardiner, 170 State st., Chicago, Ill.

ward and slightly upward, and in the left eye almost exactly outward; and, after reviewing the literature on the subject, D'O. arrives at the following conclusions:

1. Ectopia of the lens is a malformation, the causes of which, thus far, remain unknown.

2. It always affects both eyes, generally in a symmetrical manner.

3. The direction of the displacement is almost always either upward, upward and inward, or upward and outward.

4. The lenses are generally transparent; sometimes their size is below the mean.

5. The suspensory ligament is sometimes found, sometimes not.

6. In about one-fourth of all cases, there is myopia.

7. The position of the lenses may remain unchanged throughout life, but spontaneous dislocation may also result.

8. Heredity has been proven.

"ON QUININE AMAUROSIS." By Dr. E. Gruening, New York. —(*Arch. of Oph.*, *Vol.* X., *No.* 1.)

G. reports in detail the case of a lady (age thirty-five) who, after taking 80 grs. of quinine in thirty hours (10 gr. doses), was seized with a convulsive fit. When the attack had passed, the patient was found to be totally deaf and blind. Twenty-four hours after the attack, hearing was partially recovered, but total blindness persisted for one month, when she began to distinguish light. From this time (July 19) V. gradually improved until Sept. 23rd, when with + 3 D. her V.=$\frac{20}{20}$, and reads Snellen $1\frac{1}{2}$. "Her fields of vision were concentrically limited." On Snellen's color-chart no colors are recognized. On Dec. 28, the four fundamental colors were perceived. After excluding all causal elements known to bring on temporary amaurosis without appreciable retinal lesions, G. concludes that, "On reviewing the unequivocal cases of quinine poisoning with amaurosis (Roosa, Wecker, Voorhies, Gruening), we find a remarkable congruence in their essential features." "The patient, after the ingestion of a single dose, or of repeated doses of quinine, in varying quantities, suddenly becomes totally blind and deaf. While the deafness disappears within twenty-four hours, the blindness remains permanent as regards peripheric vision, central vision gradually returning to the normal after some days, weeks or months. The ophthalmoscope reveals an ischæmia of the retinal arteries and veins, without any inflammatory changes."

"In view of the constancy of these symptoms, and the uniformity of the ophthalmoscopic picture, we are entitled to demand for this type of amaurosis a recognized position in the pathology of the optic nerve and the retina."

AMBLYOPIA FROM DISUSE. By Dr. E. Williams.—(*Cin. Lan. and Clin.*)

W. advises tenotomy as soon as the strabismus is a "fixed thing," in order to save the sight of the eye, with faulty fixation. In support of the theory of amblyopia from disuse, he reports the case of a man of sixty-three years, who in boyhood had been operated upon for convergent strabismus. The effect had been excessive, the result being a very unsightly divergence, with a great insufficiency of the interni. He had learned to fix exclusively with his right eye; the left was defective in vision from "disuse, previous amblyopia, or both." Suddenly a large hæmorrhage occurred in the region of the macula of the right eye, which reduced his formerly good V to $\frac{3}{200}$. The habit of fixing with his right eye adhered to him, and when he looked with it he saw but little; when this eye was closed, and he could fix with his left eye, he saw much better. On uncovering the right eye, he involuntarily fixed with it; the left eye diverged, he suppressed the image, and was left in obscurity. By months of patient practice, he has acquired control over the muscles of his left eye, and now fixes with it. The eye was hypermetropic $\frac{1}{14}$, with +14 $V=\frac{15}{40}$; six weeks later, $V=\frac{20}{30}$. He yet sees badly at night, and can only read for a few moments at a time.

CONTRIBUTIONS TO THE STATISTICS OF MYOPIA. By Dr. O. Just, of Zittau, Saxony.

In order to ascertain whether the bad hygienic conditions of schools were the sole cause of myopia in children, J. examined seven classes of the two new High Schools of Zittau, in both of which the hygienic conditions of light, space and arrangements left nothing to be desired. Not finding the percentage decreased, he concludes that "myopia being as frequent in the new schools of Zitttau as in other educational institutions of the same kind, we may fairly conclude that it does not chiefly result from insufficient illumination of the school rooms, but rather from the great and ever-increasing demands on the industry of the pupils at home, forcing prolonged labor on their eyes during the evening hours, frequently by insufficient artificial light."

J. does not underate the dangers of bad sanitary conditions in school rooms, but urges the necessity to disencumber children's eyes of as much evening work as possible.

"DE L'AMBLYOPIE ALCOOLIQUE." By Dr. H. Romiee (de Liège).—(*Rec. D'Oph.*, Jan., 1881.)

R. considers this disease much more common than it is supposed to be. Among the most prominent symptoms he mentions the loss of accommodative power, which ranges from slight "weakness" all the way to total paralysis. *Vision* rapidly diminishes, terminating in complete blindness.

*Color perception* diminishes from the center (central scotoma), and gradually attains the periphery. *Ophthalmoscope.*—The changes in the flundus are limited to the optic nerve, the modifications of which he resumes in : (*a*) hyperæmia, (*b*) a characteristic white appearance of the papilla, (*c*) gray atrophy. After stating the general symptoms and treatment, he reports several very interesting cases.

PYÆMIA FOLLOWING A MASTOID ABSCESS, Treated without Medicine. Recovery. By E. F. Ely, New York.—(*Arch. of Ot., Vol. X., No.* 1.)

E. gives in detail a case in which, after chronic suppuration of both middle ears for many years, a mastoid abscess formed on the right side. Wilde's incision was made, and after considerable trouble a soft spot in the bone was detected. The fistula was enlarged, a poultice applied, hot douche ordered to be used. The patient apparently improved, and was considered out of danger, when suddenly, on the sixth day after the operation, he had a chill, and the temperature at 9 A. M. was 104½°. For eleven days the patient presented well-marked symptoms of pyæmia. Both the author and Dr. Roosa gave a very unfavorable prognosis to the family. During this illness no medicine was administered. "Aside from the matter of drugs, this boy, of course, had a great deal of medical treatment, in the best sense of the words. He had a quiet room to himself, with an open fire. . . . Good nursing. . . . I visited him often myself, and every small detail regarding food, stimulants, dressings, etc., received thoughtful consideration. . . . Food was well born during the entire period. . . The diet consisted of milk, to which was added a little sherry wine at first, and afterward a little whisky. Poultices were kept applied over the jugular vein and upon the painful swelling over the left sterno-clavicular joint."

"Jan. 31.—Pains and tenderness along each clavicle. A red and tender swelling, about the size of a walnut, has appeared over the left sterno-clavicular articulation. Distinct sense of fluctuation."

"Feb. 12.—There were no unfavorable symptoms after this date. . . . He went out, Feb. 26 (thirty-six days after operation). Free discharge from mastoid, fistula and ear."

"THE COTTON PELLET AS AN ARTIFICIAL DRUM HEAD." By Dr. H. Knapp, New York.—(*Arch. of Ot.*, *Vol.* X., *No.* 1.)

After giving a short historical review of the artificial drum head, K. reports four cases in which its application rendered decided services to the patient. In Case I, a lady of forty-one, who had copious and offensive discharge following scarlet fever, and who at the age of twelve was so deaf as to necessitate people to speak loud directly into her ears, was treated by Dr. F. A. Cadwell, who put cotton pellets into her ears. He attended her himself until the discharge had almost disappeared. "Since then (29 years) she has worn the cotton pellets, and by their aid has always enjoyed good hearing, and has been free from pain and inflammation." At present, without the pellets, she understands conversation at the distance of a few feet—with them at twenty. In Cases II, III, and IV, the results obtained were also excellent. The points which Dr. K. desires to make in regard to the use of the cotton pellets are summarized in the following statements quoted from his article:

1. "Cotton pellets, moistened with glycerine and water (1:4), and worn as artificial drum-heads, are a great aid to hearing in many cases of partial or total defect of the natural drum-head, with or without otorrhœa."

2. "Their therapeutical action in arresting profuse discharge on the one hand, and preventing the mucous membrane of the drum cavity from drying up, on the other, is most valuable."

3. "They protect, like natural drum-heads, the deeper parts of the ear against injurious influences of the atmosphere."

4. "In some cases they are quite indispensable, and may be worn for a lifetime with permanent comfort and benefit."

5. "In other cases they are needed only periodically, according to the copiousness of the discharge, or the exsiccation of the mucous membrane requires their action, in the one or other direction."

6. "The period during which a pellet may be left in the ear varies with the condition of the parts. They should be changed frequently—*i. e.*, every day, or every few days, so long as the discharge is considerable. They should not be worn at all when the discharge is abundant and offensive. When there is no discharge they may be left in as long as they are comfortable and the hearing is good. So far as my experience goes, they are apt to become unclean in a week or two. They then ought to be removed, the ear cleansed, either with dry cotton, or cotton steeped in warm soap-suds, and new pellets introduced."

7. "The management of the ear disease should remain in the hands of the physician until a stationary condition, either of slight or no discharge, has been reached. During the time the patient is under treatment, he can be taught how to cleanse his ear, and remove and replace the pellets."

